# A comparison of all-cause and HIV cause-specific mortality among children under 5 years of age before and during COVID-19 in Kenya, 2018–2022

**DOI:** 10.1371/journal.pgph.0004338

**Published:** 2025-05-07

**Authors:** Susan Gachau, Victor Akelo, Angela Cleveland, Joyce Were, Sammy Khagayi, Daniel Kwaro, Miriam Taegtmeyer, David Obor, Aggrey Igunza, Stephen Munga, Richard Omore, Thomas Misore, George Aol, Dickens Onyango, Beth A. Tippett Barr, Rachael Joseph

**Affiliations:** 1 Division of Global HIV&TB (DGHT), Global Health Center, US Centers for Disease Control and Prevention (CDC), Kisumu, Kenya; 2 Liverpool School of Tropical Medicine, Liverpool, United Kingdom; 3 Kenya Medical Research Institute, Center for Global Health Research, Kisumu, Kenya; 4 Kisumu County Department of Health, Kisumu, Kenya; 5 Nyanja Health Research Institute, Salima, Malawi; PLOS: Public Library of Science, UNITED STATES OF AMERICA

## Abstract

The impact of the COVID-19 pandemic on pediatric mortality, including measures to ensure continuity of HIV care, is not well described in Kenya. We evaluated causes of death (COD) among decedents under 5 years of age both before and during the COVID-19 pandemic in Kenya. We analyzed Child Health and Mortality Prevention Surveillance (CHAMPS) data collected in February 2018–March 2022. We describe the proportional contribution of specific conditions in the causal chain of death among decedents aged 28 days to 59 months who underwent minimally invasive tissue (MITS) sampling, had an HIV polymerase chain reaction, and a COD determination. We also calculated all-cause and HIV cause-specific mortality rates using data from two health and demographic surveillance system (HDSS) sites in western Kenya. Results were stratified by time periods: February 2018 to February 2020, and March 2020 to March 2022. Among 269 MITS-eligible decedents, 55.8% died during the pre-COVID period. Of these, 53.7% were infants (28 days to 11 months), and 9.7% were HIV-positive. Leading causes of death for infants included malnutrition (20.5%), pneumonia (17.5%), sepsis (17.1%), and malaria (14.5%). For older children (12–59 months), the predominant causes were malaria (25.6%), malnutrition (21.1%), pneumonia (14.1%), and sepsis (13.1%). All-cause mortality rates did not differ significantly between the periods (53.9 vs. 52.8 per 1,000 live births, p=0.77), but HIV cause-specific mortality rates were significantly lower during March 2020–March 2022 compared to February 2018–February 2020 (1.2 vs. 3.1 per 1,000 live births, p=0.01). Malaria, malnutrition, pneumonia, and sepsis were the leading COD among decedents aged 28 days to 59 months enrolled in CHAMPS between February 2018 and March 2022. These findings may point to the need for urgent, focused efforts to prevent avoidable child deaths. Continued monitoring of HIV-related mortality could provide insights into the ongoing impact of the HIV program in the region.

## Introduction

Coronavirus disease 2019 (COVID-19), caused by the severe acute respiratory syndrome coronavirus 2 (SARS-CoV-2), was declared a global pandemic by the World Health Organization (WHO) on March 11, 2020, resulting in destabilizing social, economic and health status, leading to wide-reaching implications on children’s health globally [1–3]. In Kenya, the first case of COVID-19 was reported on March 12, 2020, prompting the government to implement several public health measures to reduce infections, including school closures, curfews, restrictions on travel and social gatherings, and messaging about preventative behaviors [4].

While these interventions aimed to reduce the spread of the virus, stigma and fear of contracting COVID-19, restriction of movement, and financial challenges due to job losses impacted demand, access, and delivery of essential and routine health services in some African countries [5,6]. Moreover, the potential indirect impacts of mitigation measures on routine pediatric health services such as childhood vaccinations, HIV and malaria diagnosis and treatment, and nutritional support remain of great concern [2,7–10].

To ensure continuity and access to care among persons living with HIV (PLHIV), several measures were also implemented, including decentralization of medication pick-up sites, scale-up of multi-month dispensing (MMD) of antiretroviral therapy (ART) and TB preventative therapy (TPT), integration of tuberculosis (TB) and COVID-19 screening and diagnosis services, and virtual tracking of clients with treatment interruption [11]. Maternal and child health services measures implemented included the scale-up of MMD among pregnant and breastfeeding mothers (PBFW), virtual follow-up of mother-baby pairs, and home-based services for early infant diagnosis (EID) among infants who may have been exposed to HIV [12,13].

Sub-Saharan Africa (SSA) suffers from disproportionately high child mortality, primarily attributed to preventable childhood diseases, including pneumonia, diarrhea, and malaria [14,15]. In Kenya, the Child Health and Mortality Prevention Surveillance (CHAMPS) network was established in 2015 to conduct high-quality surveillance for child mortality and determine the causes of under-five deaths [16]. The CHAMPS surveillance system is nested within a larger health and demographic surveillance system (HDSS) in western Kenya [17,18], a region burdened with a high prevalence of HIV [19,20] and malaria [21]. We analyzed CHAMPS data on children under five, together with HDSS population surveillance system data, to compare conditions in the causal chain of death, all-cause mortality rates, and HIV cause-specific mortality rates among children under five before (February 2018-February 2020) and during the COVID-19 pandemic (March 2020-March 2022) in western Kenya.

## Methods

### Surveillance population

The Kenya CHAMPS network is a longitudinal study ongoing since 2017 in two sites in western Kenya: Karemo, a rural area in Siaya County, and Manyatta, an urban informal setting in Kisumu County, both covering a population of approximately 170,000 persons under surveillance (Karemo population: ~90,000, Manyatta: ~80,000) [16–18]. These sites are nested within a larger health and demographic surveillance system (HDSS) platform operated by the Kenya Medical Research Institute (KEMRI), which conducts surveillance across five locations, including the two CHAMPS sites of Karemo sub-county in Siaya County and Manyatta sub-county in Kisumu County. The infrastructure and population monitoring capabilities of this HDSS have been previously described [17,22]. Briefly, the HDSS enumerates all residents of a geographically defined catchment area and longitudinally monitors demographic characteristics (e.g., age, birth, and death rates, in- and out-migrations), information on access and utilization of health care services, disease outbreaks, and causes of death. Causes of death are assessed through verbal autopsies using a standardized WHO questionnaire [23,24]. Regular visits are conducted to all households in the HDSS catchment areas for demographic and health census [22]. The HDSS also conducts routine reviews and updates of data related to events, such as deaths, that may have been missed during previous censuses.

### Data collection in CHAMPS

CHAMPS data collection is described in detail elsewhere [16]. In brief, CHAMPS collects under-five mortality data  through multiple sources, including facility- and community-based mortality notification systems, verbal autopsy with caregivers, and abstraction of clinical records and anthropometric measures for nutritional assessment [17,18]. Data  on maternal factors are also collected in cases of stillbirth or infant death . Additional consent is obtained for deaths notified within 24 hours for minimally invasive tissue sampling (MITS) to collect further laboratory data [25]. Thus, a decedent enrolled in CHAMPS is considered MITS eligible if death is notified within 24 hours, not yet buried, cremated, or embalmed and guardian/parental consent is granted for the procedure. For cases that are eligible for MITS, the specimens collected undergo testing through histopathology, HIV polymerase chain reaction (PCR), and TB tests using Gene Xpert [16,18]. CHAMPS does not audit prior data collection and has a timeline for enrolling and performing MITS on deaths in children under 5 years and stillbirths (deaths detected within 24 hours, and not yet buried, cremated, or embalmed), and conducts no retrospective data collection on deaths that may have been missed. However, data cleaning for already enrolled cases continues until all required data elements are ready to be presented to the adjudication committee for the Determination of Cause of Death (DeCoDe) panel.

### Cause of death determination in CHAMPS

The final cause of death is determined by an adjudication panel consisting of a pathologist, pediatrician, neonatologist, epidemiologist, and microbiologist (referred to as a Determination of Cause of Death [DeCoDe] panel) [16]. The DeCoDe panel reviews individual case files containing all available data about the clinical course, including the child’s clinical data, anthropometric measures, laboratory results, verbal autopsy and pathology findings, and maternal data, where applicable. Immediate, intervening, and underlying causes are categorized based on World Health Organization guidance as follows: immediate causes (conditions that directly preceded or directly caused death), intervening (conditions that occurred as part of the causal chain leading to death), and underlying (conditions which triggered the series of events leading to death). Other conditions not in the causal chain are captured as other significant factors contributing to death [16]. The DeCoDe panel determines the cause of death based on available data, acknowledging that not all cases will have complete information. They use international coding standards and grade causes from 1-5 for certainty [26]. The panel reaches a consensus, noting any disagreements, and records individual assignments. Site-based panels review all cases, while a central panel in Atlanta reviews a subset of cases to ensure accuracy and consistency [16]. CHAMPS data were accessed for research purposes on March 18, 2023.

### Health demographic surveillance system (HDSS) data

We included data from under-five death notifications, and the number of live births recorded in the Karemo and Manyatta HDSSs and collected between February 2018 through March 2022. HDSS data were accessed for research purposes on May 10, 2023.

### COVID -19 data

In addition to CHAMPS and HDSS data, we also included COVID-19 data reported between March 2020 and March 2022 by the Kenyan Ministry of Health [27]. We reviewed monthly COVID-19 case counts and reported COVID-19 deaths across all groups identified in Kisumu and Siaya counties, where the HDSS and CHAMPS data were collected. COVID-19 data FOR Kisumu and Siaya Counties were accessed for research purposes on August 20, 2023.

### Data analysis

**Fig 1:**
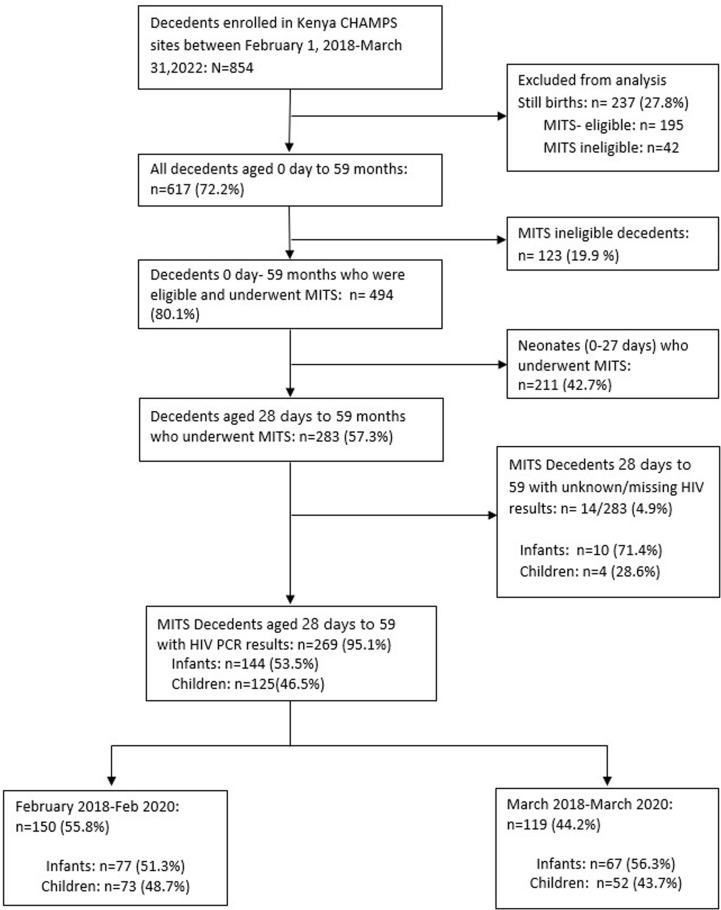
Summary of analytic sample drawn from decedents enrolled in the Kenya CHAMPS site February 1,2018– March 31, 2022. Infants: - 28 days to 11 months, children: - 12 months to 59 months.

We analyzed HDSS live births and deaths events, COVID-19 case count data, and CHAMPS child mortality data reported between February 2018 and March 2022. The analysis was stratified into two time periods: period 1 (February 1, 2018, to February 29, 2020), and period 2 (March 1, 2020, to March 31, 2022). The analysis of CHAMPS data consisted of two components, as outlined in this section. In general, we focused on decedents aged 0 days to 59 months. Our first objective was to describe the demographic characteristics of children and mothers, as well as the conditions in the causal chain of death. To achieve this, we concentrated our analyses on decedents enrolled in CHAMPS who were aged 28 days to 59 months (infants and children), eligible for, and underwent, MITS procedures (hereafter referred to as “MITS-eligible decedents”), and had a postmortem HIV test result (Fig 1). Decedents aged 28 days to 59 months who did not undergo the MITS procedure were excluded due to insufficient information regarding the underlying, immediate, and intervening conditions necessary for determining the cause of death. Additionally, neonates (0–27 days), including those who underwent MITS, were excluded because the causes of death in this age group are primarily related to birth complications [25].

Frequencies and proportions were used to describe child and maternal demographic characteristics, stratified by period. Pearson’s chi-square and Fisher’s exact tests compared proportions between time periods. Previous studies have shown that HIV is in the causal chain in nearly all deaths among HIV-positive children [18]. For comparability, frequencies and proportions of causes of death in the causal chain (immediate cases, intervening, and underlying) were therefore stratified by period and HIV status (HIV-positive vs. HIV-negative), and the frequency of occurrence of HIV disease in the causal chain of death was not analyzed. The Pearson Chi-square test was used to examine differences disease-specific prevalence by period, or the Fisher exact test was used if the expected frequency in one cell of the contingency table was less than 5.

Deaths reported in CHAMPS, along with births and deaths from the Karemo and Manyatta HDSS sites, were visualized graphically. Additionally, COVID-19 cases across all age groups reported in Siaya and Kisumu counties were depicted graphically, and case fatality rates were calculated to illustrate the impact of the pandemic in these study areas. Line charts were utilized to examine the prevalence of conditions in the causal chain of death over time.

For the second objective, which aimed to calculate all-cause and HIV cause-specific mortality rates, we analyzed data from decedents aged 0 days to 59 months who were enrolled in CHAMPS during the study period, irrespective of their MITS eligibility and HIV status. We also incorporated annual live birth data from the Karemo and Manyatta HDSS catchment sites, within which CHAMPS is embedded. All-cause and HIV cause-specific mortality rates were calculated both overall and stratified by age groups (0 days to 11 months and 12 months to 59 months) as well as by time period. To determine the under-five all-cause mortality rate per 1,000 live births, we divided the total number of death notifications for children aged 0 days to 59 months by the total number of live births reported in the two HDSS catchment sites.

To calculate overall HIV cause-specific mortality rates, we estimated the number of deaths among children with HIV in the causal chain leading to death relative to the total number of live births reported in the Karemo and Manyatta HDSS sites. The computation of mortality rates by time period accounted for incomplete calendar years. In period 1, we included the number of live births reported from February to December 2018, the total live births reported in 2019, and those reported in January and February 2020. For Period 2, we aggregated the number of live births reported from March to December 2020, the total for 2021, and those reported from January to March 2022.

We compared mortality rates across two distinct periods (February 2018 - February 2020 and March 2020 - March 2022) and by individual calendar years (2018–2021) using a Poisson regression model, as appropriate. This model was employed to estimate the expected mortality rates for each time period, adjusting for variations in the number of live births. All analyses were performed using R software (version 4.1.2), with an alpha of 0.05 for all statistical tests.

### Consent and ethical considerations

The HDSS was approved by the institutional review board of KEMRI’s Scientific Ethical Review Unit (SSC# 1801). The HDSS study was also reviewed and approved by the CDC (IRB# 3308), and its implementation was consistent with applicable federal law and CDC policy.^.^ For CHAMPS verbal autopsy data, parents provided written informed consent for participation of their deceased child in CHAMPS mortality surveillance procedures. For HDSS live birth data, the head of each household/compound gave their consent for their family to take part in the data collection activities. All the data were de-identified, and the authors did not have access to information that could identify individual participants during data collection and analysis.

## Results

Between February 1, 2018 and March 31, 2022, a total of 854 decedents under 5 years were enrolled in the Kenyan CHAMPS sites. Out of these, 27.8% (237/854) were stillbirths and were excluded from the analysis. Of the remaining 617 (72.2%) decedents aged 0 day- 59 months, 80.1% (494/617) were eligible and underwent minimally invasive tissue sampling (MITS). Among MITS-eligible decedents, infants and children aged between 28 days and 59 months accounted for 57.3% (283/494), while neonates accounted for the remaining 42.7% (211/494). Approximately 4.9% (14/283) of the decedents between 28 days and 59 months who underwent MITS procedure had a missing or unknown HIV PCR result, with infants making up about 71.4% (10/14) of those cases (Fig 1). About 95.1% (269/283) of the MITS-eligible decedents aged 28 days to 59 months had an HIV test result documented, with infants accounting for 53.5% (144/269) (Fig 1).

### Demographic, clinical and maternal characteristics of minimally invasive tissue sampling (MITS) eligible decedent children aged 28 days to 59 months enrolled in the Kenya Child Health and Mortality Prevention Surveillance (CHAMPS)

While the overall number of enrolled decedents aged 28 days to 59 months varied by time period (February 2018- February 2020: 55.8% (150/269) vs. March 2020-March 2022: 44.2% (119/269) the proportion of enrolled decedents in either Karemo or Manyatta site did not vary by time (p=0.93, Table 1).

**Table 1:** Demographic, clinical and maternal characteristics of minimally invasive tissue sampling (MITS) eligible decedent children aged 28 days to 59 months enrolled in the Kenya Child Health and Mortality Prevention Surveillance (CHAMPS) program, February 2018-March 2022.

Characteristics	February 2018- February 2020 n=150, (column %)	March 2020-March 2022 n=119, (column %)	Total N=269, (column %)	p-value
**Age category**	**n=150**	**n=119**	**n=269**	
28 days-11 months	77 (51.3)	67 (56.3)	144 (53.5)	0.38
12–59 months	73 (48.7)	52 (43.7)	125 (46.5)	
**Sex**	**n= 149**	**n=119**	**n=268**	
Female	70 (47.0)	52 (43.7)	122 (45.5)	0.58
Male	79 (53.0)	67 (56.3)	146 (54.5)	
**HDSS catchment area**	**n=150**	**n=119**	**n=269**	
Karemo	78 (52.0)	63 (52.9)	141 (52.4)	0.93
Manyatta	72 (48.0)	56 (47.1)	128 (47.6)	
**Place of death**	**n=150**	**n=119**	**n=269**	
Community	65 (43.3)	43 (36.1)	108 (40.1)	0.26
Facility	85 (56.7)	76 (63.9)	161 (59.9)	
**Number of living siblings**	**n=85**	**n=64**	**n=149**	
0	25 (29.4)	19 (29.7)	44 (29.5)	0.85
1-2	41 (48.2)	33 (51.5)	74 (49.7)	
≥3	19 (22.4)	12 (18.8)	31 (20.8)	
**HIV status based on PCR test results**	**n=150**	**n=119**	**n=269**	
HIV-negative	130 (86.7)	113 (95.0)	243 (90.3)	0.02
HIV-positive	20 (13.3)	6 (5.0)	26 (9.7)	
**ART uptake**	**n=20**	**n=6**	**n=26**	
HIV-positive, on ART	6 (30.0)	1 (16.7)	7 (26.9)	0.99
HIV-positive, not on ART	14 (70.0)	5 (83.3)	19 (73.1)	
**Maternal age**	**n=88**	**n=68**	**n=156**	
<25 years	40 (45.4)	31(45.5)	71 (45.5)	0.98
25–34 years	35 (39.8)	34 (50.0)	69 (44.2)	
>34 years	13 (14.8)	3 (4.5)	16 (10.3)	
**Maternal marital status**	**n=71**	**n=66**	**n=137**	
Currently married	59 (83.1)	55 (83.3)	114 (83.2)	0.52
Currently unmarried	12 (16.9)	11 (16.7)	23 (16.8)	
**Maternal Education**	**n=85**	**n=68**	**n=153**	
Primary or none	59 (69.4)	43 (62.0)	102 (66.7)	0.50
Secondary and above	26 (30.6)	25 (38.0)	51 (33.3)	
**Maternal HIV status**	**n=150**	**n=119**	**n=269**	
HIV-positive	35 (23.3)	14 (11.8)	49 (18.2)	0.03
HIV-negative	73 (48.7)	50 (42.0)	123 (45.7)	
Unknown	42 (28.0)	55 (46.2)	97 (36.1)	
**Maternal ART uptake**	**n=35**	**n=14**	**n=49**	
HIV-positive, on ART	26 (74.3)	10 (71.4)	36 (73.5)	0.99
HIV-positive, not on ART	9 (25.7)	4 (28.6)	13 (26.5)	
**Maternal Maternal antenatal clinic visits**	**n=64**	**n=50**	**n=114**	
1–3	33 (51.6)	25 (50.0)	58 (50.9)	0.86
≥4	31 (48.4)	25 (50.0)	56 (49.1)	

A greater proportion of deaths among decedents aged 28 days to 59 months were identified within healthcare facilities versus the community (59.9% vs 40.1%); the proportion of deaths in healthcare facilities increased from 56.7% to 63.9% between the two time periods, although the difference was not statistically significant (p=0.26). The proportion of decedents did not vary by age category (28 days to 11 months and 12–59 months) or sex between time periods (age category: p=0.38, sex: p=0.58).

Overall, 9.7% (26/269) of the enrolled decedents were HIV-positive (10 infants and 16 children), and the prevalence differed significantly between time periods (February 2018- February 2020: 13.3% [20/150] vs. March 2020- March 2022: 5.0% [6/119], p=0.02). Of the 26 HIV positive decedents, only 7 (26.9) were on ART: 30.0% (6/20) in February 2018- February 2020 and 16.7% (1/6) in March 2020-March 2022.

The majority of the decedents’ mothers were married (83%, 114/137), and 66.7%,(102/153) had primary or no education at all. Nonetheless, there was no variation in maternal marital status and education level between the two time periods. The proportion of mothers reporting HIV positive status decreased (23.3% [35/150] to 11.8% [14/119]), while the proportion of mothers reporting unknown HIV status increased (28.0% [42/150] to 46.2% [55/119], p=0.03).

**Fig 2:**
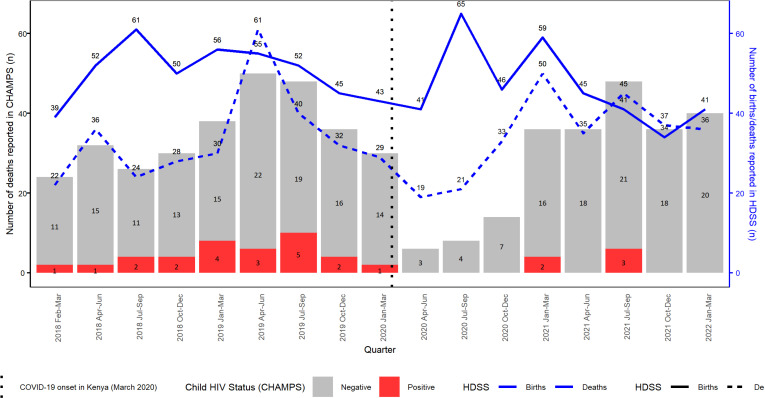
Number of deaths reported in the Kenya Child Health and Mortality Prevention Surveillance (CHAMPS) surveillance program (bar chart) and the health and demographic surveillance system (HDSS) surveillance system (line chart) among minimally invasive tissue sampling (MITS) eligible decedents aged 28 days to 59 months: February 1, 2018, to March 31, 2022.

Fig 2 shows deaths reported by quarter using both CHAMPS (n=269 decedents aged 28 days to 59 months) and HDSS data (n= 551 decedents aged 28 days to 59 months) notified during the study periods, as well as the number of births reported in the HDSS (n=825 overall). During the study period, the number of births identified in HDSS was consistently higher than the number of deaths, except for April-June 2019 and July-December 2021. The death reports in both surveillance systems exhibited a similar pattern, although death notifications in HDSS consistently exceeded those in CHAMPS. In 2018–2020, most death notifications in both CHAMPS and HDSS occurred in the second quarter (April-June) and third quarter (July-September) of 2019, followed by a gradual decline in the fourth quarter (October-December) of 2019 and the first quarter (January-March) of 2020. Within CHAMPS, there was at least one HIV-positive decedent reported in every quarter of the 2018–2020 period, with a peak in the third quarter of 2019 (Fig 2).

A drop in death notifications in both CHAMPS and HDSS was observed between April and June 2020, which coincided with the timing of the onset of the COVID-19 pandemic in Kenya (Fig 2). The drop was followed by an incremental increase in the identification of deaths in the subsequent months of 2020, however, the number of decedents reported in each quarter of 2020 was consistently lower compared to those reported in previous years (2018 and 2019). The number of recorded births in April-December 2020 remained at pre-pandemic levels. During 2021 and the first quarter (January-March) of 2022, the number of decedents enrolled in CHAMPS and those reported in HDSS site were comparable to those reported during the 2018–2020 period. There were 6 HIV positive decedents reported in CHAMPS from March 2020- March 2022.

### COVID-19 cases in Kisumu and Siaya counties

**Fig 3:**
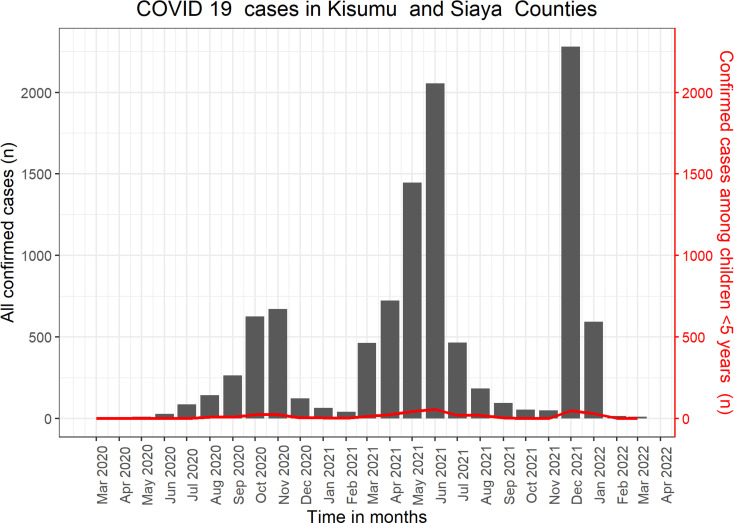
Number of confirmed COVID-19 cases over time in Kisumu and Siaya counties, Western Kenya (bar chart), and case counts among children below 5 years (line chart), enrolled in the Kenya Child Health and Mortality Prevention Surveillance (CHAMPS) program from February 1, 2018, to March 31, 2022.

Between March 2020 and March 2022, 10,496 confirmed COVID-19 cases across all age groups were reported in the CHAMPS study counties: 55.3% (5,807/10,496) in Kisumu County and 44.7% (4,689/10,496) in Siaya County. Overall, 3.3% (341/10,496) of cases were children under 5 years. The overall case fatality rate in the catchment area was 1.5% (159/10,496); 40% of reported COVID-related deaths occurred in Kisumu County (64/159) and 60% in Siaya County (95/159). Overall, 1.3% (2/159) of all COVID deaths occurred among children under 5 years. There were three waves of the pandemic in the two study counties, with peaks of infection in November 2020, June 2021, and December 2021 (Fig 3). A similar trend was observed among children under 5, although the numbers were few (Fig 3, line chart).

### Causes of death among MITS-eligible decedents 28 days to 59 months

Overall, 88.9% (239/269) of MITS-eligible decedents aged 28 days to 59 months with an HIV PCR result had a DeCoDe COD determination (February 2018- February 2020 era: 60.7%, n=145/239 vs. March 2020-March 2022: 39.3%, n=94/239). Of the 239 decedents with a COD, 90 (37.7%) had one condition, 98 (41.0%) had two conditions, 44 (18.4%) had three, and 7 (2.9%) had four or more conditions recorded. In total, 433 conditions were identified in the causal chain of death (as either underlying, intervening or immediate condition) during the study period (February 2018–2020: 57.7%, n=250/433 vs. March 2020–2022: 42.3%, n=183/433). Overall, the most frequently assigned conditions in the causal chain were malnutrition (20.8%, 90/433), malaria (19.6%, 85/433), pneumonia (15.9%, 69/433), and sepsis (15.2%, 66/433). Cumulatively, these four conditions accounted for 71.6% (310/433) of the burden of illnesses in the causal chain of death. The burden of malaria was significantly higher in Karemo compared to Manyatta HDSS site irrespective of period (p<0.05, S1, S2 Tables). On the other hand, the burden of pneumonia was significantly higher in Manyatta compared to Karemo (p <0.05, S1, S2 Tables). There were no deaths during the study period for which COVID-19 was assigned in the causal chain of death.

### Causes of death among MITS-eligible decedents aged 28 days to 11 months (infants)

Among infant decedents aged 28 days to 11 months, 87.5% (126/144) with an HIV PCR had a DeCoDe COD determination (February 2018- February 2020: 56.3%, 71/126 vs. March 2022- March 2022: 43.7%, 55/126, p = 0.04). A total of 234 conditions were assigned in the causal chain leading to death among 126 infant decedents (2018–2020: 52.6%, 123/234 vs. 2020–2022: 47.4, 111/234, p= 0.26). The most prevalent conditions in the causal chain of death (as either underlying, immediate, or intervening condition) were malnutrition (20.5%, 48/234), pneumonia (17.5%, 41/234), sepsis (17.1%, 40/234), and malaria (14.5%, 34/234). The four illnesses accounted for 69.7% (163/234) of all the conditions in the causal chain of death.

During February 2018-February 2020, malnutrition (19.5%, 24/123) was most common, followed by pneumonia (17.1%, 21/123), sepsis (16.3%, 20/123) and malaria (16.3%, 20/123). Among HIV-negative decedents, malnutrition (19.5%, 22/113), sepsis (16.8%, 19/113), and malaria (16.8%, 19/113) were most common, while pneumonia (30.0%, 3/10) was most common among HIV-positive decedents.

During March 2020- March 2022, malnutrition (21.6%, 24/111) was most common, followed by pneumonia (18.0%, 20/111) and sepsis (18.0%, 20/111) and malaria (12.6%, 14/111) (Table 2). Among HIV-negative infants, malnutrition (21.7%, 23/106) was most prevalent, while sepsis (40.0%, 2/5) was most common among HIV-positive decedents.

**Table 2:** Prevalence of illnesses in the causal chain leading to death among minimally invasive tissue sampling (MITS)-eligible decedents, with cause of death determination at the Kenyan Child Health and Mortality Prevention Surveillance (CHAMPS) catchment sites: February 2018-March 2022.

Conditions	February 2018- February 2020			March 2020- March 2022			p-value	Overall
	HIV-negative, n (column %)	HIV-positive, n (column %)	Total, n (column %)	HIV-negative, n (column %)	HIV-positive, n (column %)	Total, n (column %)		
**Infant decedents** **(28 days -11 months)**	**113**	**10**	**123**	**106**	**5**	**111**	**–**	
Malnutrition	22 (19.5)	2(20.0)	24 (19.5)	23 (21.7)	1 (20.0)	24 (21.6)	0.69	48 (20.5)
Pneumonia	18 (15.9)	3 (30.0)	21 (17.1)	19 (17.9)	1 (20.0)	20 (18.0)	0.84	41 (17.5)
Sepsis	19 (16.8)	1 (10.0)	20 (16.3)	18 (16.9)	2 (40.0)	20 (18.0)	0.72	40 (17.1)
Malaria	19 (16.8)	1 (10.0)	20 (16.3)	14 (13.2)	0 (0.0)	14 (12.6)	0.43	34 (14.5)
Gastroenteritis	8 (7.1)	1 (10.0)	9 (7.3)	10 (9.4)	0 (0.0)	10 (9.0)	0.63	19 (8.1)
Aspiration pneumonia	7 (6.2)	1 (10.0)	8 (6.5)	3 (2.8)	0 (0.0)	3 (2.7)	0.22	11 (4.7)
Prematurity	5 (4.4)	0 (0.0)	5 (4.1)	6 (5.6)	0 (0.0)	6 (5.4)	0.76	11 (4.7)
Other conditions	15 (13.3)	1 (10.0)	16 (13.0)	13 (12.3)	1 (20.0)	14 (12.6)	0.93	30 (12.8)
**Child decedents** **(12 months - 59 months)**	**100**	**27**	**127**	**65**	**7**	**72**	**–**	
Malaria	27 (27.0)	6 (22.2)	33 (26.0)	16 (24.6)	2 (28.6)	18 (25.0)	0.87	51 (25.6)
Malnutrition	17 (17.0)	10 (37.0)	27 (21.3)	13 (20.0)	2 (28.6)	15 (20.8)	0.94	42 (21.1)
Sepsis	11 (11.0)	4 (14.8)	15 (11.8)	9 (13.8)	2 (28.6)	11 (15.3)	0.48	26 (13.1)
Pneumonia	16 (16.0)	3 (11.1)	19 (15.0)	8 (12.3)	1 (14.2)	9 (13.5)	0.63	28 (14.1)
Aspiration pneumonia	6 (6.0)	1 (3.7)	7 (5.5)	2 (3.1)	0 (0.0)	2 (2.8)	0.99	9 (4.5)
Gastroenteritis	3 (3.0)	2 (7.4)	5 (3.9)	4(6.2)	0 (0.0)	4 (5.6)	0.72	9 (4.5)
Other conditions	20 (20.0)	1 (3.7)	21 (16.5)	13 (20.0)	0 (0.0)	13 (18.1)	0.78	34 (17.1)

### Causes of death among MITS-eligible decedents aged 12 months to 59 months (children)

Among children decedents aged 12–59 months, 90.4% (113/125) with an HIV PCR result had a COD determination (February 2018- February 2020: 65.5%, n = 74/113 vs. March 2022- March 2022: 34.5%, 39/113, p <0.001).

A total of 199 conditions were assigned in the causal chain leading to death as either underlying, intervening or immediate condition. Overall, malaria (25.6%, 51/199) was the most common, followed by malnutrition (21.1%, 42/199), pneumonia (14.1%, 28/199), and sepsis (13.1, 26/199). These four illnesses accounted for 73.9% (147/199) of all the conditions in the causal chain of death.

During February 2018- March 2020, malaria was the most prevalent condition (26.0%, 33/127), followed by malnutrition (21.3%, 27/127), and pneumonia (15.0%, 19/127). Similar proportions were observed among HIV-negative decedent children. Among HIV positive decedent children, malnutrition (37.0%, 10/27), malaria (22.2%, 6/27), and sepsis (14.8%, 4/27) were most common (Table 2).

During March 2020- March 2022, the top four conditions in the causal chain of death were malaria (25.0%, 18/72), malnutrition (20.8%, 15/72), sepsis (15.3%, 11/72), and pneumonia (13.5%, 9/72). Similar proportions were observed among HIV-negative decedents. Among HIV positive children (n=72), malaria (25%, 18/72), malnutrition (20.8%, 15/72), and sepsis (15.3%, 11/72) were most common.

**Fig 4:**
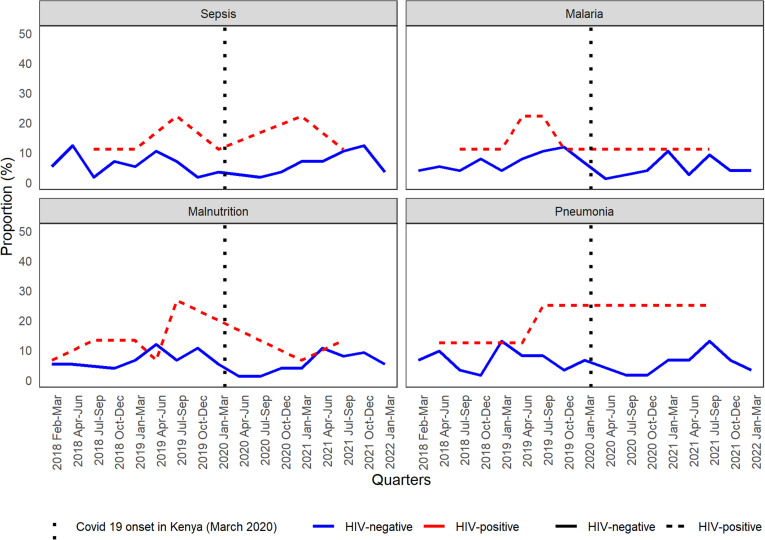
Prevalence of sepsis, malaria, malnutrition, and pneumonia in the causal chain of death among minimally invasive tissue sampling (MITS)-eligible decedents aged 28 days to 59 months enrolled in the Kenya Child Health and Mortality Prevention Surveillance (CHAMPS) sites from February 1, 2018, to March 31, 2022.

Fig 4 represents prevalence trends in the top four conditions in the causal chain of death (i.e., sepsis, malaria, pneumonia, and malnutrition) among decedents aged 28 days to 59 months. Prevalence fluctuated throughout the study period for both HIV-positive and HIV-negative decedents. Nonetheless, the prevalence of specific conditions in the causal chain was consistently higher among HIV-positive decedents (Fig 4).

### All-cause and HIV cause-specific mortality rates

The under-5 all-cause mortality rate decreased from 53.9 deaths per 1,000 live births during February 2018–February 2020 to 52.8 deaths per 1,000 live births during March 2020–March 2022; however, Poisson regression analysis did not provide strong evidence of a statistically significant difference between the two periods (p = 0.75, Table 3). In contrast, the under-5 HIV cause-specific mortality rate decreased significantly from 3.1 deaths per 1,000 live births to 1.2 deaths per 1,000 live births (p = 0.006).

**Table 3:** Under-five years and age-stratified all-cause and HIV cause-specific mortality rates per 1,000 live births by time periods, Kenya Child Health and Mortality Prevention Surveillance (CHAMPS) program, February 2018-March 2022.

Mortality indicator	February 2018-February 2020	March 2020- March 2022	p-value
Under-five years mortality rate (0 day to 59 months)			
Number of under-five years deaths notified to HDSS	483	478	–
HIV prevalence among children in CHAMPS	5.9	2.2	–
Estimated number of deaths caused by HIV	27	10	–
Number of live births	8958	9047	–
Under-five years mortality rate per 1,000 live births	53.9	52.8	0.75
HIV-cause specific rate per 1,000 live births	3.1	1.2	0.006
Neontes and Infant (0 day to 11 months) mortality rate			
Number of infant deaths notified to HDSS	342	347	–
HIV prevalence among infants in CHAMPS	3.4	4.4	–
Estimated number of deaths caused by HIV	10	10.1	–
Number of live births	8958	9047	–
Infant mortality rate per 1,000 live births	38.2	38.4	0.95
HIV-cause specific rate per 1,000 live births	1.2	1.1	0.99
Children (12 months to 59 months) mortality rate			
Number of deaths notified to HDSS	141	131	–
HIV prevalence among children in CHAMPS	12.7	7	–
Estimated number of deaths caused by HIV	16.8	6.6	–
Number of live births	8958	9047	–
Mortality rate per 1,000 live births	15.7	14.5	0.49
HIV-cause specific rate per 1,000 live births	1.9	0.73	0.03

When stratified by age, all-cause mortality rates were generally higher among neonates and infants (0 days to 11 months) compared to children (12 months to 59 months) during February 2018–February 2020 (neonates/infants: 38.2 deaths per 1,000 live births vs. children: 15.7 deaths per 1,000 live births, p < 0.001). A similar pattern was observed during March 2020–March 2022 (38.4 deaths per 1,000 live births among neonates and infants vs. 14.7 deaths per 1,000 live births among children, p < 0.001). HIV-specific mortality rates did not differ significantly between neonates/infants and children in both time periods (p > 0.05).

Among neonates and infants, all-cause mortality rates (February 2018–February 2020: 38.2; March 2020–March 2022: 38.4, p = 0.95) and HIV-specific mortality rates (February 2018–February 2020: 1.2; March 2020–March 2022: 1.1, p = 0.99) did not vary significantly between time periods (Table 3).

Among children, all-cause mortality rates remained stable (February 2018–February 2020: 15.7; March 2020–March 2022: 14.7, p = 0.49), while the HIV cause-specific mortality rate decreased significantly (February 2018–February 2020: 1.9 deaths per 1,000 live births; March 2020–March 2022: 0.7 deaths per 1,000 live births, p = 0.03).

The under-5 all-cause mortality rate varied significantly by individual year between 2018 and 2021 (S3 Table), with rates ranging from 61.8 deaths per 1,000 live births in 2019 to 46.1 deaths per 1,000 live births in 2020. Poisson regression analysis revealed that the under-5 all-cause mortality rate in 2019 was significantly higher compared to 2018 (p = 0.02). In contrast, 2021 showed a trend toward increased mortality relative to 2018, but this result was marginally significant (p = 0.07). While there was a decrease in the under-5 all-cause mortality rate in 2020 compared to 2018, this decrease was not statistically significant (p = 0.4). Similarly, the HIV cause-specific mortality rate significantly varied by year, decreasing from 4.5 deaths per 1,000 live births in 2019 to 1.0 deaths per 1,000 live births in 2020 (p = 0.01, S3 Table).

## Discussion

In Kenya, malaria, malnutrition, and pneumonia were the top contributors to the causal chain of death among HIV-positive and-negative decedents aged 28 days to 59 months who underwent MITS in CHAMPS between February 2018 and March 2022. Additionally, we observed no significant difference in all-cause mortality between the two periods; however, there was a notable decrease in HIV-cause specific mortality among children aged 12–59 months during 2020–2022 period.

The number of decedents enrolled in the Kenyan CHAMPS sites and HDSS varied between time periods, with a significant drop that began in April-June 2019 (prior to COVID-19 reaching Kenya) and continued into mid-2020, followed by a subsequent increase. These fluctuations may be attributable to several factors, including shifts in disease transmission dynamics (such as malaria and respiratory diseases) and the implementation of sanitation and social distancing measures to combat COVID-19 (including handwashing, mask mandates, school closures, and the use of hand sanitizer), which effectively reduced the spread of common childhood illness, and subsequently lowered childhood mortality rates [28].

While the overall proportion of death notifications reported in either community or health care facilities did not vary by time periods, about 40% of the MITS-eligible deaths occurred in communities during both study periods. This suggests suboptimal utilization of health care facilities and potentially delayed health care-seeking behavior among caregivers of sick children. These caregivers may be opting for community-based care or facing barriers to accessing healthcare, leading to delays in obtaining appropriate medical attention. Furthermore, although not statistically significant, there was a slight increase in the proportion of deaths occurring within healthcare facilities from 56.7% to 63.9% between the two time periods.

Nearly 10% of decedents were HIV-positive during the study period, however, the number and prevalence of HIV-positive decedents was significantly fewer during the pandemic period compared to the pre-COVID-19 period. This may be a consequence of both the rapid scale-up of ART by the national HIV program and efforts to ensure access to ART during COVID-19 pandemic. In 2015, the Kenya Ministry of Health adopted universal treatment of HIV within the national HIV program, rapidly scaling up ‘test-and-treat’ thereafter [29]. Consequently, estimated coverage of antiretroviral therapy (ART) of the adult population (15–49 years) in Siaya and Kisumu counties increased from approximately 80% (76% among men, >82% among women) in 2018 to over 97% in both males and females in 2022 [30]. Over 95% of women in Siaya and Kisumu counties report accessing antenatal clinics at least once during their most recent pregnancy (KENPHIA 2018 full report) [31]; over the study period (2018– 2022), estimated coverage of ART prior to ANC attendance increased from 73% to 88% in Siaya and 70% to 83% in Kisumu [31]. However, these study results revealed that the proportion of mothers with decedent children aged 28 days to 59 months with unknown HIV status increased from 28% to 46% between the two periods. This suggests that although efforts were made to ensure access to antiretroviral therapy (ART) for those already on treatment, measures implemented to control the spread of COVID-19 may have unintentionally impacted uptake and utilization of HIV services. For instance, physical distancing, limitations on movement, stigma, and fear of contracting COVID-19 may have affected the demand, access, and delivery of essential routine health services, including HIV testing services [32].

Almost 10% of descendants were HIV-infected during the study period, however, the number and prevalence of HIV-infected decedents was significantly fewer during the pandemic compared to the pre-COVID-19 period. Despite the disruptions to global health service delivery and concerns about challenges accessing care for HIV services during the pandemic, the impact on HIV care and treatment outcomes in Kenya was well-mitigated,with the national HIV treatment cohort expanding during this time [33]. This resilience is attributed to the adaptability of Kenya’s HIV program, supported by various programmatic interventions implemented at the onset of the pandemic. These measures included accelerated efforts to stockpile key commodities at the county level, the introduction of three- to six-month multi-month ART dispensing, the promotion of flexible ART delivery models (e.g., community groups for ART distribution to decongest health facilities), and improved virtual coordination and supervision of HIV service delivery [12,13,33,34].

Four preventable and treatable illnesses—malaria, malnutrition, pneumonia, and sepsis—collectively accounted for 71.6% of the burden of illnesses leading to death among children aged 28 days to 59 months. We observed that the prevalence of the top four conditions in the causal chain of death varied over time. From February 2018- February 2020, malaria was the most common cause overall and among HIV-negative decedents, while malnutrition predominated among HIV-positive decedents. Notably, the burden of malaria was higher in Karemo compared to Manyatta HDSS site, regardless of age group. However, from March 2020–March 2022, malnutrition emerged as the leading cause of death irrespective of HIV status. These findings align with previous studies conducted in western Kenya, which found malaria and malnutrition to be the leading causes of death (COD) among children aged 1–59 months [18].

Further analysis by age revealed that malnutrition was the most frequently assigned cause of death among infants throughout the study period, whereas malaria dominated in the causal chain of death among decedents aged 12 months to 59 months. Notably, the most recent national HIV survey in Kenya revealed that among children 6 months to 5 years, Siaya County had a prevalence of severe malnutrition at 2.7%, which is ten times higher than the national estimate of 0.2%. Additionally, the prevalence of moderate malnutrition was also high at 4.1%, four-times higher than the national estimate of 0.9% [35]. These findings highlight a persistent health disparity that could potentially be addressed through improved access to nutritional support for infants and children in these regions. Our study findings are consistent with the national survey, highlighting the importance of focused measures to further prevent morbidity and mortality related to malnutrition among both HIV-positive and -negative children. In 2018, the Siaya county government, in collaboration with stakeholders, initiated an action plan modeled after the National Nutrition Action Plan to combat malnutrition [36]. These efforts represent a crucial step toward mitigating this preventable cause of childhood mortality and reducing health disparities in the region; however, continued monitoring may be needed to assess the impact of these interventions on child health and mortality.

In western Kenya, malaria transmission is perennial and high with seasonal peaks occurring in June-July and November-December, despite high reported usage of long-lasting insecticidal nets [21]. Previous studies have indicated that malaria infection prevalence is highest among children aged 5–14 years, yet children <5 years of age are much more likely to have adverse outcomes from infection [21,37]. However, there is optimism that child mortality will decrease significantly following the recent rollout of the RTS,S vaccine in Kenya. This vaccine has shown promising results, with a 10% reduction in all-cause mortality among children in areas where the vaccine was administered, compared with areas that it was not [38].

We found that children under 5 years represented only 3.25% of the confirmed COVID-19 cases. The SARS-COV-2 PCR testing in CHAMPS was performed retrospectively, analyzing samples collected in March 2020- March 2022. Interestingly, despite this, none of the decedents enrolled during the study period had COVID-19 in the causal chain of death. These findings align with recent reports suggesting differences in susceptibility or severity of COVID-19 infection among children compared to adults [39].

The under-five years all-cause mortality rate in CHAMPS did not vary between time periods, but an assessment of individual years revealed variations with small upsurges in 2019 and 2021. Even so, mortality rates in Kisumu and Siaya are above the national average [40]. This could be explained by the geographical location of CHAMPS catchment sites in western Kenya, a region characterized by high malaria transmission and HIV prevalence and consequently higher mortality rates [23].

Additionally, we observed higher all-cause mortality rates among decedent aged 0 day to 11 months compared with children aged 12 months to 59 months. This finding underscores the importance of public health surveillance systems specifically geared toward addressing infant mortality. Infants remain an especially vulnerable population requiring targeted interventions to promote timely detection and appropriate management of common childhood illnesses and further reduce preventable deaths. In this study, the level of ART uptake among HIV-positive decedents was poor, despite widespread HIV testing and ART access. Additionally, there was an increase in the proportion of mothers with decedents ages 28 days-59 months with unknown status between the two study periods. These observations could be attributed to possible program gaps, including missed testing and diagnoses, as well as patient-related factors such as treatment interruptions.

This study was subject to several limitations. First, in Kenya, the CHAMPS study is implemented in two HDSS sites in western Kenya, and disease patterns and causes of death among children under 5 may not be representative of the rest of the country. Secondly, we analyzed routinely collected data with missing information for maternal age, marital status, education status, and ANC2 visits. This could potentially underestimate or overestimate the differences in proportions across time periods. Thirdly, we analyzed data retrospectively, and were therefore unable to ascertain whether the decline in enrollment of MITS eligible cases at the onset of the COVID-19 pandemic was due to an actual decrease in deaths or under reporting of deaths; however, the steep decline in deaths preceding March 2020 suggests other factors were also at play. Nonetheless, death notifications in CHAMPS followed a similar pattern to that observed in HDSS (although numbers in HDSS were much higher compared to those in CHAMPS) and further evaluation would be useful to understand the factors behind the declines. Lastly, we focused our comparison on overall prevalences due to the low number of deaths among children with HIV, which limited our ability to detect statistical differences in causes between HIV-positive and HIV-negative children.

## Conclusion

Malaria, malnutrition, pneumonia, and sepsis were the leading COD among decedents aged 28 days to 59 months who underwent MITS in CHAMPS between February 2018 and March 2022. Malaria was most prevalent during February 2018-February 2020, while malnutrition led during March 2020- March 2022. The under 5-all-cause mortality rates did not differ between time periods but HIV-cause specific mortality rate was significantly lower during March 2020-March 2022. These findings underscore the importance of targeted efforts to prevent avoidable child deaths, particularly from malaria and malnutrition. Our findings also signal a promising decrease in HIV-associated mortality; continued monitoring of HIV-related mortality is critical to assess the ongoing impact of the HIV program in the region.

## Supporting information

S1 Table Prevalence of illnesses in the causal chain leading to death among MITS-eligible decedents, with cause of death determination at Karemo CHAMPS catchment site in Kenya between February 2018- March 2022. (DOCX)

S2 Table Prevalence of illnesses in the causal chain leading to death among MITS-eligible decedents, with cause of death determination at Manyatta CHAMPS catchment site in Kenya between February 2018 and March 2022. (DOCX)

S3 Table Under-five mortality rate and HIV cause-specific mortality rate per 1, 000 live births in the Kenya CHAMPS site: 2018–2021. (DOCX)

S1 Data CHAMPS and HDSS Data. (ZIP)
