# Supplementary material for: A comparison of all-cause and HIV cause-specific mortality among children under 5 years of age before and during COVID-19 in Kenya, 2018–2022
Source: PLOS Glob Public Health. 2025 May 7;5(5):e0004338. doi: 10.1371/journal.pgph.0004338 (PMC12058159; doi:10.1371/journal.pgph.0004338)
Supplement: S1 Table — (DOCX) [file pgph.0004338.s001.docx]

S1 Table: Prevalence of illnesses in the causal chain leading to death among MITS-eligible decedents, with cause of death determination at Karemo CHAMPS catchment site in Kenya between February 2018- March 2022.

|  | **February 2018- February 2020** | | | **March 2020-March2022** | | |  |
| --- | --- | --- | --- | --- | --- | --- | --- |
| **Illnesses/conditions in the causal chain leading to death** | HIV-uninfected,  n (column %) | HIV-infected,  n (column %) | Total,  n (column %) | HIV-uninfected,  n (column %) | HIV-infected,  n (column %) | Total,  n (column %) | p-value^1^ |
| **All decedents**  **(28 days to 59 months)** | 112 | 16 | 128 | 94 | 8 | 102 | **-** |
| Malaria | 38 (33.9) | 6 (37.5) | 44 (34.4) | 25 (26.6) | 2 (25.0) | 27 (26.5) | 0.19 |
| Malnutrition | 23 (20.5) | 5 (31.2) | 28 (21.9) | 20 (21.3) | 2 (25.0) | 22 (21.6) | 0.96 |
| Pneumonia | 15 (13.4) | 2 (12.5) | 17 (13.3) | 11 (11.7) | 0 (0.0) | 11 (10.8) | 0.56 |
| Sepsis | 13 (11.6) | 1 (6.2) | 14 (10.9) | 14 (14.9) | 4 (50.0) | 18 (17.6) | 0.14 |
| Gastroenteritis | 1 (0.9) | 1 (6.2) | 2 (1.6) | 6 (6.4) | 0 (0.0) | 6 (5.9) | 0.68 |
| Aspiration pneumonia | 5 (4.5) | 1 (6.2) | 6 (4.7) | 1 (1.1) | 0 (0.0) | 1 (1.0) | 0.13 |
| Prematurity | 2 (1.8) | 0 (0.0) | 2 (1.6) | 3 (3.2) | 0 (0.0) | 3 (2.9) | 0.14 |
| Other conditions | 15 (13.4) | 0 (0.0) | 15 (11.7) | 14 (14.9) | 0 (0.0) | 14 (13.7) | 0.64 |
| **Infant decedents**  **(28 days -11 months)** | 46 | 5 | 51 | 54 | 3 | 57 | **-** |
| Malnutrition | 10 (21.7) | 1 (20.0) | 11 (21.6) | 12(22.2) | 1(33.3) | 13(22.8) | 0.87 |
| Pneumonia | 7 (15.2) | 1 (20.0) | 8 (15.7) | 8(14.8) | 0 | 8(14.0) | 0.80 |
| Sepsis | 6 (13.0) | 0 (0.0) | 6 (11.8) | 8(14.8) | 2(66.7) | 10(17.5) | 0.39 |
| Malaria | 14 (30.4) | 1 (20.0) | 15 (29.4) | 12(22.2) | 0(0.0) | 12 (21.1) | 0.31 |
| Gastroenteritis | 1 (2.2) | 1 (20.0) | 2 (3.9) | 5(9.3) | 0(0.0) | 5 (8.8) | 0.44 |
| Aspiration pneumonia | 1 (2.2) | 1 (20.0) | 2 (3.9) | 1(1.9) | 0(0.0) | 1 (1.8) | 0.45 |
| Prematurity | 2 (4.3) | 0 (0.0) | 2 (3.9 | 3(5.6) | 0(0.0) | 3 (5.3) | 0.44 |
| Other conditions | 5 (10.9) | 0 (0.0) | 5 (9.8) | 5(9.3) | 0(0.0) | 5 (8.8) | 0.44 |
| **Child decedents**  **(12 months - 59 months)** | 66 | 11 | 77 | 40 | 5 | 45 | **-** |
| Malaria | 24(36.4) | 5(45.5) | 29(37.7) | 13(32.5) | 2(40.0) | 15(33.3) | 0.63 |
| Malnutrition | 13(19.7) | 4(36.4) | 17(22.1) | 8(20.0) | 1(20.0) | 9(20.0) | 0.78 |
| Sepsis | 7(10.6) | 1(9.1) | 8(10.4) | 6(15.0) | 2(40.0) | 8(17.8) | 0.24 |
| Pneumonia | 8(12.1) | 1(9.1) | 9(11.7) | 3(7.5) | 0(0.0) | 3(6.7) | 0.53 |
| Aspiration pneumonia | 4(6.1) | 0(0.0) | 4(5.2) | 0(0.0) | 0(0.0) | 0(0.0) | 0.30 |
| Gastroenteritis | 0(0.0) | 0(0.0) | 0(0.0) | 1(2.5) | 0(0.0) | 1(2.2) | 0.78 |
| Other conditions | 10(15.2) | 0(0.0) | 10(13.0) | 9(22.5) | 0(0.0) | 9(20.0) | 0.30 |

p-value^1^: comparing proportions of illnesses/conditions in the causal chain leading to death pre and during COVID-19 period.
