# Supplementary material for: A comparison of all-cause and HIV cause-specific mortality among children under 5 years of age before and during COVID-19 in Kenya, 2018–2022
Source: PLOS Glob Public Health. 2025 May 7;5(5):e0004338. doi: 10.1371/journal.pgph.0004338 (PMC12058159; doi:10.1371/journal.pgph.0004338)
Supplement: S2 Table — (DOCX) [file pgph.0004338.s002.docx]

S2 Table: Prevalence of illnesses in the causal chain leading to death among MITS-eligible decedents, with cause of death determination at Manyatta CHAMPS catchment site in Kenya between February 2018 and March 2022.

|  | **February 2018- February 2020** | | | **March 2020-March 2022** | | |  |
| --- | --- | --- | --- | --- | --- | --- | --- |
| **Illnesses/conditions in the causal chain leading to death** | HIV-uninfected,  n (column %) | HIV-infected,  n (column %) | Total,  n (column %) | HIV-uninfected,  n (column %) | HIV-infected,  n (column %) | Total,  n (column %) | p-value^1^ |
| **All decedents**  **(28 days to 59 months)** | 101 | 21 | 122 | 77 | 4 | 81 | **-** |
| Malaria | 8 (7.9) | 1(4.8) | 9(7.4) | 5 (6.5) | 0(0.0) | 5(6.2) | 0.74 |
| Malnutrition | 16(15.8) | 7(33.3) | 23(18.9) | 16 (20.8) | 1(25.0) | 17 (21.0) | 0.71 |
| Pneumonia | 19(18.8) | 4(19.0) | 23 (18.9) | 16 (20.8) | 2 (50.0) | 18 (22.2) | 0.83 |
| Sepsis | 17(16.8) | 4(19.0) | 21(17.2) | 13(16.9) | 0(0.0) | 13 (16.0) | 0.55 |
| Gastroenteritis | 10(9.9) | 2(9.5) | 12 (9.8) | 8(10.4) | 0(0.0) | 8 (9.9) | 0.99 |
| Aspiration pneumonia | 8(7.9) | 1(4.8) | 9 (7.4) | 4(5.2) | 0(0.0) | 4 (4.9) | 0.56 |
| Prematurity | 3(3.0) | 0(0.0) | 3 (2.5) | 3(3.9) | 0(0.0) | 3 (3.7) | 0.98 |
| Other conditions | 20 (19.8) | 2(9.5) | 22 (18.0) | 12(15.6) | 1 (25.0) | 13 (16.0) | 0.71 |
| **Infant decedents**  **(28 days -11 months)** | 67 | 5 | 72 | 52 | 2 | 54 | **-** |
| Malnutrition | 12 (19.9) | 1(20.0) | 13 (18.1) | 11(21.2) | 0(0.0) | 11 (20.4) | 0.74 |
| Pneumonia | 11(16.4) | 2(40.0) | 13(18.1) | 11(21.0) | 1(50.0) | 12(22.2) | 0.56 |
| Sepsis | 13(19.4) | 1(20.0) | 14(19.4) | 10(19.2) | 0(0.0) | 10 (18.5) | 0.89 |
| Malaria | 5(7.5) | 0(0.0) | 5(6.9) | 2(3.8) | 0(0.0) | 2(3.7) | 0.69 |
| Gastroenteritis | 7(10.4) | 0(0.0) | 7(9.7) | 5(9.6) | 0(0.0) | 5 (9.3) | 0.99 |
| Aspiration pneumonia | 6(9.0) | 0(0.0) | 6(8.3) | 2(3.8) | 0(0.0) | 2 (3.7) | 0.46 |
| Prematurity | 3(4.5) | 0(0.0) | 3(4.2) | 3(5.8) | 0(0.0) | 3(5.6) | 0.99 |
| Other conditions | 10(14.9) | 1(20.0) | 11 (15.3) | 8(15.4) | 1(50.0) | 9 (16.9) | 0.83 |
| **Child decedents**  **(12 months - 59 months)** | 34 | 16 | 50 | 25 | 2 | 27 | **-** |
| Malaria | 3 (8.8) | 1(6.2) | 4(8.0) | 3(12.0) | 0(0.0) | 3(11.1) | 0.69 |
| Malnutrition | 4 (11.8) | 6 (37.5) | 10(20.0) | 5(20.0) | 1(50.0) | 6(22.2) | 0.71 |
| Sepsis | 4 (11.8) | 3 (18.8) | 7(14.0) | 3(12.0) | 0(0.0) | 3(11.1) | 0.66 |
| Pneumonia | 8 (23.5) | 2(12.5) | 10(20.0) | 5(20.0) | 1(50.0) | 6(22.2) | 0.71 |
| Aspiration pneumonia | 2 (5.9) | 1(6.2) | 3(6.0) | 2(8.0) | 0(0.0) | 2(7.4) | 0.95 |
| Gastroenteritis | 3 (8.8) | 2(12.5) | 5 (10.0) | 3(12.0) | 0(0.0) | 3(11.1) | 0.99 |
| Other conditions | 10(29.4) | 1(6.2) | 11 (22.0) | 4(16.0) | 0(0.0) | 4(14.8) | 0.55 |

p-value^1^: comparing proportions of illnesses/conditions in the causal chain leading to death pre and during COVID-19 period.
