# Supplementary material for: A comparison of all-cause and HIV cause-specific mortality among children under 5 years of age before and during COVID-19 in Kenya, 2018–2022
Source: PLOS Glob Public Health. 2025 May 7;5(5):e0004338. doi: 10.1371/journal.pgph.0004338 (PMC12058159; doi:10.1371/journal.pgph.0004338)
Supplement: S3 Table — (DOCX) [file pgph.0004338.s003.docx]

S3 Table: Under-five mortality rate and HIV cause-specific mortality rate per 1, 000 live births in the Kenya CHAMPS site: 2018- 2021

| Mortality indicator | Year 2018 | Year 2019 | Year 2020 | Year 2021 | p -value |
| --- | --- | --- | --- | --- | --- |
| Number of under-five deaths notified to HDSS *(0 to 59 months)* | 214 | 254 | 191 | 259 | - |
| HIV prevalence among children in CHAMPS (*0 to 59 months*) (%) | *3.8* | *7.9* | *2.1* | *4.3* | *-* |
| Estimated number of under-five deaths who were HIV-infected in HDSS | 8 | 20 | 4 | 11 | - |
| Estimated number of deaths caused by HIV | 8.2 | 18.6 | 4.2 | 11.2 | - |
| Number of live births | 4253 | 4107 | 4145 | 4366 | **-** |
| Under-five mortality rate per 1,000 live births | 50.3 | 61.8 | 46.1 | 59.3 | 0.003 |
| HIV-cause specific rate per 1,000 live births | 1.9 | 4.5 | 1.0 | 2.5 | 0.01 |
